# Elliptic Fourier Analysis in the Study of the Male Genitalia to Discriminate Three *Macrolophus* Species (Hemiptera: Miridae)

**DOI:** 10.3390/insects8040120

**Published:** 2017-11-01

**Authors:** A. M. Jauset, E. Edo-Tena, P. M. Parés-Casanova, C. Castañé, N. Agustí, O. Alomar

**Affiliations:** 1 Department of Crop and Forest Sciences, University of Lleida, Alcalde Rovira Roure, 177, 25198 Lleida, Spain; eetena@gmail.com; 2 Department of Animal Production, University of Lleida, Alcalde Rovira Roure, 177, 25198 Lleida, Spain; peremiquelp@prodan.udl.cat; 3 IRTA, Ctra. Cabrils Km 2, 08348 Cabrils (Barcelona), Spain; cristina.castane@irta.cat (C.C.); nuria.agusti@irta.cat (N.A.); oscar.alomar@irta.cat (O.A.)

**Keywords:** geometric morphometrics, morphology, paramere, *Macrolophus pygmaeus*, *Macrolophus melanotoma*, *Macrolophus costalis*

## Abstract

Within the genus *Macrolophus* (Heteroptera: Miridae), the species *M. costalis* (Fieber), *M. melanotoma* (Costa) and *M. pygmaeus* (Rambur) are present in the Mediterranean region on a wide variety of plant species. While *M. costalis* can easily be separated from the other two by the black tip at the scutellum, *M. pygmaeus* and *M. melanotoma* are cryptic species, extremely similar to one another in external traits, which has resulted in misidentifications. *M. pygmaeus* is an efficient biological control agent, both in greenhouse and field crops. The misidentification of these cryptic species could limit the effectiveness of biological control programs. Although the morphology of the left paramere of the male genitalia has been used as a character for identification of these two cryptic species, there is controversy surrounding the reliability of this character as a taxonomic tool for these species. Using geometric morphometric techniques, which are a powerful approach in detecting slight shape variations, the left parameres from these three *Macrolophus* species were compared. The paramere of *M. costalis* was larger and had a different shape to that of *M. melanotoma* and *M. pygmaeus*; however, no differences in size or shape were found between the left paramere of *M. melanotoma* and that of *M. pygmaeus*. Therefore, our results confirm that this character is too similar and it cannot be used to discriminate between these two cryptic species.

## 1. Introduction

Within the genus *Macrolophus* (Heteroptera: Miridae), the species *M. costalis* (Fieber, 1858), *M. melanotoma* (Costa, 1853) and *M. pygmaeus* (Rambur, 1839) are present in the Mediterranean region on a wide variety of plant species. *Macrolophus* is the most unresolved genus of the Dicyphinae, probably polyphyletic. Palaearctic species have a very similar morphology, and do not appear to be congeneric with Western Hemisphere species [1,2]. In the case of these species, *M. costalis* can easily be separated from the other two by the black tip of the scutellum, but *M. pygmaeus* and *M. melanotoma* are cryptic species, extremely similar to one another in external traits [2], which has resulted in misidentifications [3,4]. *Macrolophus pygmaeus* is an efficient predator of several key pests of vegetable crops in Europe, and it is produced and widely used as a biological control agent, both in greenhouse and field crops [5,6,7,8]. Both species are sympatric in some of their distribution range within the Palearctic region and can share the same host plants. Failing to recognize cryptic species limits the effectiveness of the biological control programs, has economic consequences, and could cause rejection of potentially valuable species as control agents because prey breadth could be misinterpreted. In this particular case, different authors have confirmed that they are two different species using different approaches [3,4,9,10,11].

Genital structures are widely used in morphological studies of insects because they vary among species more than other structures, and much of the species diversity is characterized by differences in shapes [12], rather than size metrics [13,14]. The male paramere is a valuable and commonly used character for species identification in Hemiptera and more specifically in Miridae. In copulation, the left paramere is moved out of the paramere socket and the apical process is fixed on the female genital segment [15]. Its structure is important during mating and can contribute to reproductive isolation. However, the role of the left paramere in differentiating between *M. pygmaeus* and *M. melanotoma* has been controversial.

When Wagner [16] described *M. melanotoma* (as *M. caliginosus* [17]), he stated that the left paramere was clearly large and had a long apical process in comparison to *M. pygmaeus* that had a shorter and stronger apical process. Later, Wagner and Weber [18] described the left paramere of *M. pygmaeus* as ‘curved’, but ‘less curved’ than in *M. melanotoma* and also added drawings of both species. Tamanini [19] redrew and described the left paramere body of *M. pygmaeus* as enlarged at the basis, while that of *M. melanotoma* was oval, almost regular. He proposed using the left paramere for discrimination between both species due to their external morphological similarities. On the contrary, according to Josifov [2], the parameres of the male genitalia are similar among the different *Macrolophus* species and cannot be used as a valid identification. Martinez-Cascales et al. [9] also did not mention differences between the parameres of both species. However, both statements were not supported by any measure data or statistical analysis.

The traditional method for visualizing and recording morphological characters of insect genitalia is by bright field light microscopy [20], but this methodology does not allow to detect small structural variations. Geometric Morphometric (GM) techniques are acknowledged as a more powerful approach than traditional morphometrics in detecting and describing even slight shape variations [21] and, as a statistical analysis of shape, have been used to clarify the relationship of closely related taxa [22]. Two main types of GM are known: landmark-based methods, which analyze variation in the relative position of assigned landmarks; and outline-based methods, which analyze variation in the shapes of the outlines of structures. Among the second type, Elliptic Fourier Descriptors (EFDs) [23] can compare any type of shape with a similar contour. The principal component scores obtained can be used as values of morphological features in subsequent analysis, such as analysis of the shapes of biological organs. Elliptic Fourier Analysis (EFA) has been successfully used to analyze shape variation in a wide number of insect genitalia studies [22,24,25,26,27,28,29].

The aim of this study was to statistically confirm the statements of Josifov and Martinez-Cascales [1,8] of discriminating between the left paramere of *M. pygmaeus* and *M. melanotoma* using EFA, and to compare the results with those of *M. costalis* as a reference species.

## 2. Material and Methods

### 2.1. Samples Collection

Adults of *M. pygmaeus*, *M. melanotoma* and *M. costalis* were originally collected in a tomato crop, on *Dittricchia viscosa* Greuter (Asterales: Asteraceae), and on *Cistus albidus* L. (Malvales: Cistaceae) plants respectively near Mataró (Barcelona, NE of Spain, 41.556 North, 2.475 East). They were reared on tobacco plants and fed with *Ephestia kuehniella* Zell. (Lepidoptera: Pyralidae) eggs under controlled conditions (25 ± 1 °C, 70 ± 10% RH, and L16:D8 photoperiod) [30,31].

### 2.2. Preparation of Genitalia

In this study, 34 males of *M. pygmaeus*, 29 males of *M. melanotoma*, and 26 males of *M. costalis* were used. Individuals were taken from the rearing colonies, killed by freezing, and stored in 70° ethanol. Specimens were dissected in Beadle saline solution (128.3 mM NaCl, 4.7 mM KCl and 23 mM CaCl_2_) under a stereoscopic microscope (Leica MZ 12.5, Leica Microsystems, Wetzla, Germany) [32]. The distal part of the abdomen was clipped, placed in 10% KOH, and incubated in an oven (60 °C) for 4 h to remove soft tissues. After that, the specimen was neutralized with 5% glacial acetic acid and dehydrated in 99° ethanol. The left paramere was dissected in glycerin using a fine needle. Dissected parameres were mounted in glycerin jelly mounting media (1:17:17, gelatin:glycerin:distilled water) using coverslip spacers in order to avoid compression [20]. The rest of each specimen was individually stored in 70° ethanol.

All specimens were first identified [2], and all *M. pygmaeus* and *M. melanotoma* were also tested by conventional PCR [4] in order to double check their identification.

### 2.3. Elliptic Fourier Analysis

Dorsal digital images of each paramere were obtained under a bright field microscope (Leica DM4000B, (Leica Microsystems, Wetzla, Germany) provided with a Leica DFC300FX camera and processed with the Qwin V 3 (Leica) software (Leica Microsystems, Wetzla, Germany). Images were directly input as GIF files. Subsequently, image noise was manually removed and contours extracted using a graphics software package (Gimp Photoshop version 14.2.1.CC^®^, free software foundation, Boston, USA) and transformed to BMP-256 color files. Each color image was converted into a binary image (black and white). Outlines of male parameres were digitized for examination of shape variation using the software package SHAPE version 1.3, that contains four programs—ChainCoder, Chc2Nef, PrinComp and PrinPrint—for processing digital images, obtaining EFDs, performing principal component analysis, and visualizing shape variations explained by the principal [33]. ChainCoder reduces noise, traces the contours of objects, and describes the contour information as chaincode [34]. Elliptic Fourier transformations were used to calculate the EFDs [23]. Shape was approximated by the first 20 harmonics (H) [33], in which each harmonic corresponded to the four coefficients defining the ellipse on the xy-plane (an, bn, cosine coefficients and cn, dn, sine coefficients respectively). The first harmonic (H0) does not contain morphological information [35], so 76 ((4 × 20) − 4) standardized FDs were finally considered. The size and orientation of each contour was standardized using the Chc2Nef software program [33], with which the coefficients effectively became shape variables. These coefficients are mathematical descriptors of the shape that can then be statistically analyzed by routine methods [23].

### 2.4. Statistical Analysis

To analyze differences in paramere size, the Kruskal–Wallis test was used. When significant, the Mann–Whitney U-test was performed and the *p*-values were corrected for multiple comparisons using the Bonferroni technique.

The analysis of principal components (PCA) was used to summarize independent shape characteristics. Jollie cut-offs for the PCA eigen values were used to determine the number of principal components that significantly contributed to the variation in paramere shape. To reconstruct the outlines explained by each PC and to visualize what the individual PCs represent, we used the inverse Fourier transformation.

To test the null hypothesis that species were not significantly different, we used a nonparametric multivariate analysis of variance (NPMANOVA) with the Euclidean distance measure for all harmonics (H1 to H20). Pairwise comparisons were performed using the Bonferroni test.

Genital allometry was assessed by regressing genitalia shape against genitalia size (log transformed values). The statistical treatment was performed with the PAST Package v. 2.17c [36]. The significance level was established at 5%.

## 3. Results

**Figure 1:**
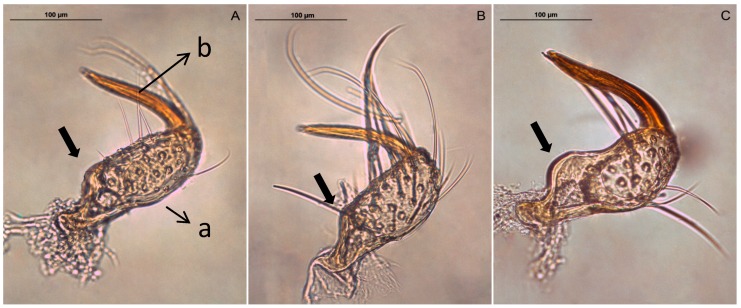
Dorsal view of the left paramere of *M. pygmaeus* (**A**), *M. melanotoma* (**B**) and *M. costalis* (**C**). Parts of the paramere: body with the sensory lobe (a) and apical process (b). The arrows show body shape differences between species.

The dorsal view of the left paramere of three *Macrolophus* species seen through the microscope is shown in Figure 1. All three species have a clearly sickle-shaped paramere, with a long apical process and a well-developed sensory lobe with elongated setae.

The paramere of *M. costalis* is larger and has a more pronounced curve in the upper side of the paramere body than in the other two species (arrow in Figure 1). Between *M. pygmaeus* and *M. melanotoma*, only slight differences are observed.

**Figure 2:**
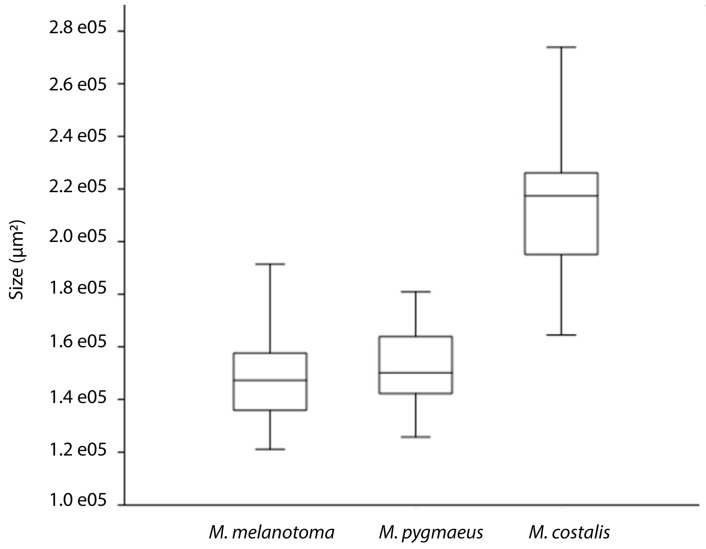
Box plot of left paramere areas (µm^2^) of *M. pygmaeus*, *M. melanotoma* and *M. costalis*. For each species, the 25–75 percent quartiles are drawn using a box. The median is shown with a horizontal line inside the box. The minimal and maximal values are shown with short horizontal lines (“whiskers”). *M. costalis* had a significantly bigger paramere than *M. melanotoma* and *M. pygmaeus*, whereas no differences were observed between the later species (*p* < 0.005).

The statistical analysis of the EFDs clearly showed differences in size (area) between parameres (*χ*^2^ = 53.83, *p* < 0.005). The paramere of *M. costalis* was the largest, whereas no differences in size were found between the parameres of *M. pygmaeus* and *M. melanotoma* (Figure 2).

Paramere outline shape variations were described by the first eight PCs that accounted for 95.24% of the total variance, with the two first PCs accounting for 75.85% of the variation (Table 1).

**Figure 3:**
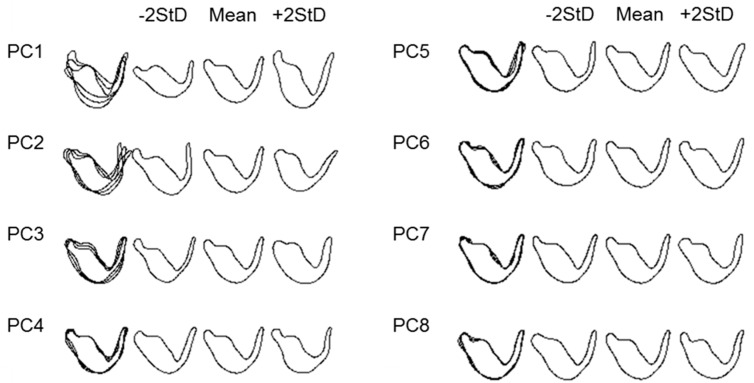
Elliptic Fourier Analysis of the dorsal view of the left paramere outlines. Reconstructions of outlines using the inverse Fourier transform based on the mean value, and the mean ± 2 s.d. of each of the first eight PCs. Overlapping lines in the first column denote areas of paramere variation.

Almost no change was observed for any reconstruction using more harmonics. The shape variation of the dorsal view of the paramere described by the eight effective PCs is illustrated in the contour reconstructions of the mean (and standard deviation) (Figure 3).

**Figure 4:**
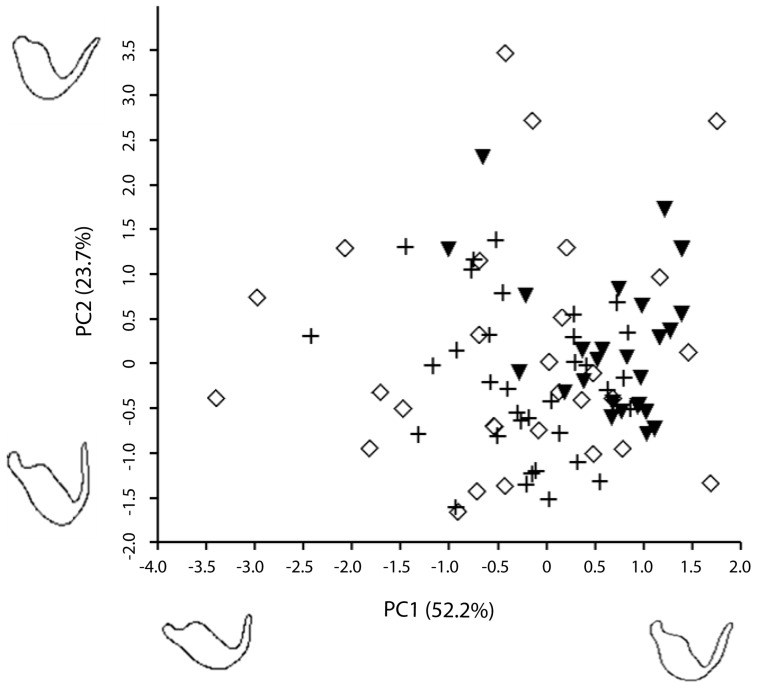
Morphological shape space of *M. pygmaeus* (+), *M. melanotoma* (◊) and *M. costalis* (▼). Each symbol corresponds to one individual.

Thus, based on these contours, it is possible to visualize that much of the shape variation described by PC1 (52.2%) is associated with the paramere body, differences in width and upper body curvature, and with the length of the apical process whereas the variation of PC2 (23.7%) is mainly associated with the angle between the apical process and the paramere body. A high PC1 value produces a characteristic body shape with a marked curvature and a long apical process, while a high PC2 value determines a greater angle between the apical process and the body of the paramere. Figure 4 illustrates the morphological space and differences in paramere shape among the three species by representing PC1 vs. PC2. *Macrolophus melanotoma* is the species that shows more variability and overlaps with all the morphological space of the other two species, whereas *M. costalis* is the species with the least variability.

The NPMANOVA reflected differences between species according to shape (*F* = 5.54, *p* = 0.0002): *M. costalis* differed in the shape of the left paramere from *M. pygmaeus* (*F* = 12.25, *p* = 0.0003) and *M. melanotoma*, (*F* = 6.90, *p* = 0.0015), whereas no significant differences were found between the paramere of *M. pygmaeus* and *M. melanotoma* (*F* = 0.87, *p* > 0.05).

The regression of genitalia sizes and shapes revealed that there was no allometry (R^2^ = 0.01, Wilk’s λ = 0.75, *p* = 0.45), so the shape of the paramere does not seem to change depending on the different sizes found in the different examined individuals. Therefore, the left paramere of *M. costalis* was larger and had a different shape to those of *M. melanotoma* and *M. pygmaeus*; however, no differences in sizes or shapes of the left paramere were found between *M. melanotoma* and *M. pygmaeus*.

## 4. Discussion

The methodology used in this study showed that there were no differences in the paramere morphology between *M. pygmaeus* and *M. melanotoma*, thus this genitalia trait is not useful for differentiating between these two species. These results are consistent with previous observations on the morphology of the left paramere of both species [2,9] although no data was provided to substantiate this affirmation. Conversely, the descriptions and drawings of the left paramere of *M. pygmaeus* and *M. melanotoma* [16,17,19] showed differences in the paramere shape. The results of the present work indicate that these differences were not consistent. However, the use of GM permits the separation of *M. costalis* from *M. pygmaeus* and *M. melanotoma* by means of the shape and size of the left paramere, which had not been reported before.

Integrative taxonomy has been used for the differentiation of the two cryptic species *M. pygmaeus* and *M. melanotoma* [4]. Both species have different genetic profiles [9], and can be clearly discriminated by using specific primers through conventional PCR [4]. Furthermore, the two species differ in the karyotype (2n = 28 (26 + XY) in *M. pygmaeus* and 2n = 34 (32 + XY) in *M. melanotoma*) and sperm morphology [11]. Although it has been shown that *M. melanotoma* and *M. pygmaeus* are two different species, some infertile inter-crossings have been observed [3,4]. In fact, cuticular hydrocarbon profiles that are used for sexual recognition, sequence variation of DNA, and some karyotype characteristics of *M. pygmaeus* are more similar to those of *M. costalis* than to those of *M. melanotoma* [9,10,11], suggesting that differentiation of the paramere may not be necessary to avoid inter-crossing.

There are several reasons why species boundaries might not be correlated with morphological change or might not be useful in discriminating between species. Among them, some species are differentiated by non-visual mating signals. Organisms that communicate reproductive signals via nonvisual means (for example, sound, vibration, pheromones or electrical signals) are perhaps most likely to harbor cryptic species because changes in signals conveyed in these modalities need not involve morphological change [37].

Another common supposition is that most cryptic species result from the speciation phenomena, which are so recent that morphological traits or any other traditional diagnostic characters have not yet evolved [38,39,40], being distinguishable only by means of molecular analyses [39]. In the case of *M. pygmaeus* and *M. melanotoma*, as has been mentioned above, the most probable reason for their morphological similarity is that they are differentiated by non-visual mating signals.

## Figures and Tables

**Table 1:** Shape analysis of the left paramere of *M. pygmaeus*, *M. melanotoma* and *M. costalis* showing the eight effective principal components (PCs) with the corresponding eigenvalues and proportions of variance explained. Almost no change was observed for any reconstruction using more harmonics.

PC	Eigenvalue	% Variance
1	1.14269e12	52.1680
2	5.18737e11	23.6820
3	1.54099e11	7.0352
4	1.01518e11	4.6347
5	7.52726e10	3.4365
6	3.77691e10	1.7243
7	3.15860e10	1.4420
8	2.49245e10	1.1379
**Cumulative variance (%)**		**95.24**
